# Yoga Practice Reduces the Psychological Distress Levels of Prison Inmates

**DOI:** 10.3389/fpsyt.2018.00407

**Published:** 2018-09-03

**Authors:** Anis Sfendla, Petter Malmström, Sara Torstensson, Nóra Kerekes

**Affiliations:** ^1^Department of Biology, Faculty of Sciences, Abdelmalek Essaâdi University, Tetouan, Morocco; ^2^Department of Health Sciences, University West, Trollhättan, Sweden

**Keywords:** mental health, psychological distress, prison inmates, RCT, yoga

## Abstract

**Background:** Psychiatric ill-health is prevalent among prison inmates and often hampers their rehabilitation. Rehabilitation is crucial for reducing recidivistic offending. A few studies have presented evidence of the positive effect of yoga on the well-being of prison inmates. The conclusion of those previous studies that yoga is an effective method in the rehabilitation process of inmates, and deserves and requires further attention.

**Aims:** The current study aimed to evaluate the effect of 10 weeks of yoga practice on the mental health profile, operationalized in the form of psychological distress, of inmates.

**Methods:** One hundred and fifty-two volunteer participants (133 men; 19 women) were randomly placed in either of two groups: to participate in weekly 90-min yoga class (yoga group) or a weekly 90-min free-choice physical exercise (control group). The study period lasted for 10 weeks. Prior to and at the end of the study period the participants completed a battery of self-reported inventories, including the Brief Symptom Inventory (BSI).

**Results:** Physical activity (including yoga) significantly reduced the inmates' levels of psychological distress. Yoga practice improved all primary symptom dimensions and its positive effect on the obsessive-compulsive, paranoid ideation, and somatization symptom dimensions of the BSI stayed significant even when comparing with the control group.

**Conclusions:** Yoga as a form of physical activity is effective for reducing psychological distress levels in prison inmates, with specific effect on symptoms such as suspicious and fearful thoughts about losing autonomy, memory problems, difficulty in making decisions, trouble concentrating, obsessive thought, and perception of bodily dysfunction.

## Introduction

Originally, prisons were established to isolate convicted offenders in order to keep society safe. The major tasks of these institutions included punishment and correction of individuals who often had a history of aggression and/or antisocial behavior (1). Over time, the purpose of correctional institutions changed; it shifted from isolation and punishment to prevention of relapse into criminality by using different treatment programs. As psychiatric ill-health is a common condition among prison inmates (2, 3), today's correctional institutions pay greater attention to the issue of inmates' mental health as a factor determining the risk of relapse. It has been estimated that prisons hold more than three times as many persons with psychiatric problems and/or diagnoses than can be found in psychiatric facilities (4). Personality and mood disorders, addiction, and neurodevelopmental syndromes are the most frequent mental health problems occurring in prison inmates (5, 6). Psychopathic personality profile (7), substance abuse and attention deficit hyperactivity disorder are the most recurring diagnoses when considering the risk of relapse into a criminal life style (8).

The Swedish Prison and Probation Service aims to enhance inmates' opportunities to reintegrate into society upon release and their ability to live a drug free and law-abiding life. To this end, the Prison and Probation Service requires all inmates to participate in rehabilitation activities, 6 h a day, 5 days a week. As the capabilities and needs of inmates differ greatly, there is a wide range of activities to choose from. Beside occupational activities, inmates are provided opportunities to participate in study programs, rehabilitation programs concerning drug- and alcohol abuse, violent behavior and aggression, yoga classes, health programs, drama, painting and writing classes, etc (9).

The Prison and Probation Service has offered yoga classes to inmates since 2008 [for details on the “Krimyoga” offered, please see Kerekes et al. (10)]. Yoga aims to reconstruct the balance between body and mind by using physical, psychological, and spiritual practices. The core of yoga is conscious, deep and slow breathing during which different body movements are performed, while shifting one's focus to the body in still positions (body awareness) and to one's feelings at the moment. In some yoga forms, the session ends with meditation. The physiological and psychological impacts of yoga have been tested in various samples: general population, clinical settings, and prison inmates (11–13). It has been shown that regular exercises of yoga reduce physiological response to stress (14), state and trait anxiety and depression (15), antisocial behaviors (10), as well as anger and aggression (16). Furthermore, regular yoga practice is coupled with increased positive and decreased negative emotional states (10, 17), decreased stress (17), as well as with significantly improved impulse control and sustained attention (10). Importantly, it has been shown that yoga in correctional settings has a positive effect on risk factors associated with criminal recidivism (10).

Beside the important psychological benefits, yoga practitioners experience physiological benefits, such as improved body posture and breathing technique, increased body awareness and easier relaxation (18). In terms of biological benefits, regular yoga exercise has been found to favor normalized activity of the hypothalamus, pituitary-, and adrenal glands, and the autonomic nervous system (19), to decrease the concentration of cortisol, and to increase the concentration of serotonin and melatonin (18). As yoga can be practiced in group settings, it can also have positive effects in terms of social relations and feelings of belonging among those practicing yoga together (20).

Although the effects of yoga have already been studied, only a few studies have been conducted among prison inmates. The aim of this study is to investigate the effect of 10 weeks of yoga practice on the participating prison inmates' mental health profile, operationalized in the form of psychological distress levels.

## Subjects and methods

### Procedure and sample

The present study is part of a broader research, therefore for a detailed description of the study procedure and methods, please refer to Kerekes et al. (10). In short, they were as follows: During a 20-month period in 2013, 2014, and 2015, male and female inmates from several high- and medium-security class prisons participated in the study. After giving their consent to participate, the inmates were randomly placed in either of two groups: a group participating once a week for 10 weeks in a 90-min long yoga class (the yoga group); a group participating once a week for 10 weeks in a 90-min long free-choice non-yoga physical exercise (the control group). Prior to the start (Time 1) of the 10-week study period and again upon its completion (Time 2), the participants completed a survey, comprising several self-reported measures (assessing stress, aggression, affective states, sleep-quality, and psychological well-being). One of these measures was the Brief Symptom Inventory, which assesses a person's level of psychological distress.

A total sample of 226 inmates (201 men; 25 women) participated in the study. The average attrition rate was 32.7%. For detailed description of attrition rates and reasons in the yoga and control groups, please see Kerekes et al. (10). The final number of participants completing the whole study period was 152 (133 men; 19 women). The participants were evenly distributed between the yoga group and the control group [yoga group: 77 (67 male and 10 female) with a mean age of 36.4 years; control group: 75 (66 male and 9 female) with a mean age of 34.9 years]. A detailed description of the participants' characteristics, including comparison between the yoga and the control groups was previously published in Kerekes et al. (10), where participants in the yoga and control groups were comparable in gender distribution, age, length of sentence, and security level of holding prison.

### Instrument—the brief symptom inventory

The Brief Symptom Inventory (BSI) is a self-reported measure of psychological distress and psychiatric symptoms (21). The inventory consists of 53 items, assessing 9 primary symptom dimensions (see details below), and the Global Severity Index (GSI), which measures the individual's overall psychological distress level. Participants are asked to rate how much a problem has bothered or distressed them over the previous month. Items can be rated on a five-point Likert scale from 0 to 4 (0 = not at all, 1 = a little, 2 = moderately, 3 = substantially, and 4 = extremely). Higher scores therefore indicate greater psychological distress, whereas lower scores indicate better psychological well-being and/or psychiatric health. The 9 primary symptom dimensions are as follows:

1. Anxiety: Indications of tension, restlessness, and nervousness, as well as experiences of free-floating anxiety or panic. The following items defined the Anxiety scale in the present study:
  “Nervousness or shakiness inside,” “suddenly scared for no reason,” “feeling fearful,” “feeling tense or keyed up,” “spells of terror or panic,” “feeling so restless you could not sit still.”
2. Depression: A wide range of symptoms and signs from dysphoric affect and mood, loss of energy and interest in life activities to feelings of hopelessness and uselessness. The following items defined the Depression scale in the present study:
  “Thoughts of ending your life,” “feeling lonely,” “feeling blue,” “feeling no interest in things,” “feeling hopeless about the future,” “feelings of worthlessness.”
3. Interpersonal Sensitivity: Feelings of inadequacy and inferiority and signs of uneasiness and discomfort during interpersonal interactions. The following items defined the Interpersonal Sensitivity scale in the present study:
  “Your feelings are easily hurt,” “feelings that people are unfriendly or dislike you,” “feeling inferior to others,” “feeling very self-conscious with others.”
4. Hostility: Feelings of irritability and annoyance, the presence of frequent arguments and urges to break things. The following items defined the Hostility scale in the present study:
  “Feeling easily annoyed or irritated,” “temper outbursts that you cannot control,” “having urges to beat, injure, or harm someone,” “having urges to break or smash things,” “getting into frequent arguments.”
5. Obsessive-Compulsive: Thoughts and actions that are experienced as irresistible and persistent. The following items defined the Obsessive-Compulsive scale in the present study:
  “Trouble remembering things,” “feeling blocked in getting things done,” “having to check and double check what you do,” “difficulty in making decisions,” “your mind going blank,” “trouble concentrating.”
6. Psychoticism: Psychoticism is described as a continuum pending between a mildly alien life style and extremely psychotic status. Outside the psychiatric population, this dimension can measure social alienation. The following items defined the Psychoticism scale in the present study:
  “The idea that someone else can control your thoughts,” “feeling lonely even when you are with people,” “never feeling close to another person,” “the idea that something is wrong with your mind.”
7. Paranoid Ideation: Suspicious, hostile, and fearful thoughts of losing autonomy. The following items defined the Paranoid Ideation scale in the present study:
  “Feeling that others are to blame for most of your troubles,” “feeling that most people cannot be trusted,” “feeling that you are watched or talked about by others,” “others not giving you proper credit for your achievements,” “feeling that people would take advantage of you if you let them.”
8. Phobic Anxiety: Phobias such as fear of crowds, open spaces, conveyances, etc. The following items defined the Phobic Anxiety scale in the present study:
  “Feeling afraid in open spaces,” “feeling afraid to travel on buses, subways, or trains,” “having to avoid certain things, places or activities because they frighten you,” “feeling uneasy in crowds,” “feeling nervous when you are left alone.”
9. Somatization: The perception of bodily dysfunction. Aches, pains, and other discomforts stemming from the musculature or complaints coupled to the cardiovascular, gastrointestinal, and respiratory systems. The following items defined the Somatization scale in the present study:
  “Faintness or dizziness,” “pains in heart or chest,” “nausea or upset stomach,” “trouble getting your breath,” “numbness or tingling in parts of your body,” “feeling weak in parts of your body,” “feeling heavy in arms or legs.”

### Yoga classes and free-choice physical exercise

The yoga classes were led by prison officers who had received training in yoga instruction provided by the Swedish Prison and Probation Service. The yoga classes (“Krimyoga”) were based on *hatha yoga*, a form of physical yoga that includes elements of relaxation specifically developed for use in correctional settings. Each yoga class comprised (in sequence) at least 10 min of warm up, a defined combination of *asanas* (yoga postures), about 5 min of breathing exercises, and finally 5 min of relaxation and deep relaxation. The weekly yoga classes lasted for 90 min and were carried out uniformly across the different correctional facilities.

Free-choice physical exercise could typically include training in gym, walking, basketball, or football. Post-intervention assessment in our study included a question on time spent on physical activity and ensured that each participant (of the control group) indeed has spent at least 90 min every week with physical activity.

### Data analysis

As the low number of female participants in the study (19 of 152) did not allow gender-specific analyses, male and female subjects' data were analyzed together in this study. Violation of normality assumption was revealed by Shapiro–Wilk test in the assessed BSI data. Non-parametric tests were therefore performed. Wilcoxon Signed Rank test was used to assess changes in BSI scores from Time 1 (beginning of the 10-week study period) to Time 2 (end of the study period) within each group (yoga and control) and Mann-Whitney *U*-test was conducted to compare differences between the groups. The effect size was calculated by dividing the z value by the square root of N (total number of cases). Cohen (22) criteria of 0.1 = small effect, 0.3 = medium effect, 0.5 = large effect were applied for the results interpretation. Significance level was defined at *p* < 0.05. The data was processed with the help of the statistical program SPSS version 23 (IBM).

### Ethics

The regional Ethical Review Board in Linköping approved the study (Dnr 2013/302 – 31). The prison inmates received complete information (written and oral) about the study and the conditions of participation. Participation was entirely voluntary and all participants were ensured that their answers would in no way affect their actual prison sentence to be served. Participation required the signature of a written consent form. The collected information was treated confidentially and the researchers received only coded data. Participants who completed the whole study received a compensation of 200 Swedish crowns (about 20 euros/US dollars).

## Results

The data analysis revealed significant changes in all but three primary symptom dimensions (obsessive-compulsive, somatization, and phobic anxiety) in the control group. These significant changes were improvements with small effect sizes (*r* varied between 0.15 and 0.29) (Table 1). In the yoga group all dimensions of psychological distress were significantly improved with small or medium effect size (*r* varied between 0.19 and 0.36) (Table 1). While the Global Severity index decreased in both groups with a similar, medium effect size, the effect size of the measured improvements in each primary dimension was always bigger within the yoga group. Table 1 summarizes the results of the within-group analyses on the General Severity Index (GSI) and the 9 primary symptom dimensions of the BSI in the control and the yoga groups separately.

**Table 1:** Changes within groups (yoga and control) measured with z-scores, means (M), and standard deviations (SD) on the global severity index and on the primary symptom dimensions at pre- and post-intervention assessment in both groups.

	**Within groups**											
	**Control group (*****n*** = **75)**						**Yoga group (*****n*** = **77)**					
**Psychological distress BSI**	**Time 1 M(SD)**	**Time 2 M(SD)**	**Average change**	***p***_within_	***z***	***r***	**Time 1 M(SD)**	**Time 2 M(SD)**	**Average change**	***p***_within_	***z***	***r***
Global severity index	1 (0.7)	0.8 (0.7)	−0.2 (0.4)	< 0.001	−3.7	0.30	0.8 (0.5)	0.6 (0.6)	−0.3 (0.5)	< 0.001	−3.8	0.30
Anxiety	1.2 (0.9)	0.9 (0.9)	−0.3 (0.7)	< 0.001	−3.62	0.29	1.1 (0.8)	0.8 (0.8)	−0.4 (0.6)	< 0.001	−4.69	0.33
Depression	1.2 (0.9)	1.0 (1.0)	−0.2 (0.8)	0.016	−2.41	0.19	1 (0.8)	0.7 (0.7)	−0.4 (0.7)	< 0.001	−4.68	0.33
Interpersonal Sensitivity	0.9 (0.9)	0.8 (1.0)	−0.1 (0.7)	0.046	−2	0.15	0.8 (0.8)	0.4 (0.6)	−0.3 (0.7)	< 0.001	−4.05	0.29
Hostility	0.9 (0.8)	0.7 (0.7)	−0.2 (0.8)	0.019	−2.34	0.18	0.8 (0.7)	0.5 (0.7)	−0.2 (0.6)	0.003	−3	0.21
Obsessive-compulsive	1.3 (0.9)	1.2 (0.9)	−0.2 (0.7)	0.057	−1.9	0.15	1.3 (0.9)	0.8 (0.7)	−0.4 (0.6)	< 0.001	−5.03	0.36
Psychoticism	0.9 (0.8)	0.7 (0.8)	−0.2 (0.6)	0.012	−2.5	0.20	0.7 (0.8)	0.5 (0.7)	−0.2 (0.7)	0.003	−2.95	0.21
Paranoid ideation	1.3 (0.9)	1 (0.9)	−0.2 (0.6)	0.005	−2.83	0.22	1.1 (0.9)	0.7 (0.7)	−0.5 (0.7)	< 0.001	−4.6	0.33
Phobic anxiety	0.6 (0.9)	0.5 (0.9)	−0.1 (0.7)	0.217	−1.23	0.09	0.6 (0.8)	0.3 (0.7)	−0.2 (0.6)	0.007	−2.7	0.19
Somatization	0.7 (0.6)	0.7 (0.6)	−0.1 (0.6)	0.114	−1.58	0.12	0.6 (0.6)	0.4 (0.6)	−0.3 (0.5)	< 0.001	−4.16	0.30

Between-group analyses revealed highly significant changes in three primary BSI symptom dimensions: obsessive-compulsive, paranoid ideation, and somatization. A significantly lower mean was reported for the score on the obsessive-compulsive dimension by the yoga group (mean rank = 62.72, *n* = 71) compared to the control group (mean rank = 82.01, *n* = 73), (*z* = −2.79, *p* = 0.005, *r* = 0.23). A significantly lower mean rank difference was also reported for the score on the paranoid ideation dimension by the yoga group (mean rank = 65.02, *n* = 71) compared to the control group (mean rank = 80.66, *n* = 74), (*z* = −2.25, *p* = 0.024, *r* = 0.18), and similarly for the primary dimension of somatization by the yoga group (mean rank = 64.46, *n* = 71) compared to the control group (mean rank = 80.32, *n* = 73), (*z* = −2.29, *p* = 0.022, *r* = 0.19). All of these differences had a small effect size. Table 2 summarizes the results of the between-group analyses on the General Severity Index (GSI) and the 9 primary symptom dimensions of the BSI.

**Table 2:** Between–group comparison on the global severity index and the primary symptom dimensions of BSI (*n* varies between 76 and 64 for the control group and between 77 and 50 for the yoga group in the different dimensions).

**Psychological distress BSI**	**Mean Rank**		***p***_between_	***z***	***r***
	**Control group**	**Yoga group**			
Global severity index	61.28	52.66	0.17	−1.38	0.12
Anxiety	76.69	68.19	0.219	−1.23	0.10
Depression	78.91	67.63	0.105	−1.62	0.13
Interpersonal sensitivity	81.55	68.18	0.055	−1.92	0.16
Hostility	77.68	74.38	0.64	−0.47	0.04
Obsessive-compulsive	82.01	62.72	0.005	−2.79	0.23
Psychoticism	75.81	72.11	0.593	−0.53	0.04
Paranoid ideation	80.66	65.02	0.024	−2.25	0.18
Phobic anxiety	77.94	69.18	0.191	−1.31	0.11
Somatization	80.32	64.46	0.022	−2.29	0.19

## Discussion

The present study provides further evidence that physical activity generally reduces psychological distress and psychiatric complaints in prison inmate samples. The main finding of the present study is that yoga practice has specific positive effects on the mental ill-health of prison inmates, offering significant help with symptoms of paranoid ideation, memory problems, trouble concentrating, obsessive thought, and somatization.

In this study, inmates were randomized into either a group where they could participate in a weekly yoga exercise or into a group where they could practice free-choice non-yoga physical activity, both during a 10-week period. Both groups of inmates reported significantly improved psychological distress levels, mostly in terms of decreased levels of anxiety, depressive symptoms, interpersonal sensitivity, suspiciousness, hostility, fearful thoughts, and social alienation.

### General effects of physical activity (including yoga) on the level of psychological distress and/or psychiatric complaints

This study revealed a significantly decreased level of anxiety and depressive symptoms in both groups. The negative association between physical activity and prevalence of depression and anxiety disorders has been repeatedly described in previous research and repeated now with our study. A few studies even examined the association between physical activity and these complaints in a prospective design. A review of these prospective studies suggests that physical activity may be clinically effective, at least in major depression and panic disorder (23). Moreover, cardiovascular and resistance training and high-intensity strength training have been found to significantly reduce depression scale scores after a 9-month testing period (24), cardiovascular and resistance training has showed significantly improved numbers in interpersonal sensitivity, and high-intensity strength training has showed improved numbers in anxiety, phobic anxiety, and hostility (24). This study supports that these measures (interpersonal sensitivity, phobic anxiety, and hostility) also significantly improve after 10 weeks of yoga exercises. Consistent with previous findings, this study also shows that yoga can be used advantageously against depression and anxiety (25, 26).

Previous research found that yoga, as a complement to drug treatment, is suitable for patients diagnosed with schizophrenia and helps patients with psychoticism or paranoid ideation to regain better quality of life and socio-professional function (26, 27). In this study, we found that these dimensions of psychological distress were significantly improved not only by yoga, but also by regular non-yoga free-choice physical activity.

### Specific effects of yoga on psychological distress and/or psychiatric complaints

In this study, we measured a significant decrease in paranoid symptoms—such as suspicious and fearful thoughts about others planning or willing to take one's autonomy—after 10 weeks of yoga classes when compared to the non-yoga control group. This is in line with previous findings from clinical studies where yoga therapy programs revealed significant improvement of both positive and negative psychotic symptoms in schizophrenia patients (28). This study is, however, the first to show a reduction of paranoid thoughts in prison inmates.

The other primary symptom dimension for which a significant difference was measured between the yoga group and the control group after the study period was the obsessive-compulsive symptom dimension. The effect of yoga particularly on obsessive-compulsive disorder was suggested in previous research (29). Furthermore, in a clinical population an increased effect of pharmaceutical treatment was observed when patients with obsessive-compulsive symptoms participated in yoga classes (30).

Yoga has a direct calming effect and can lead to an improved ability to control thoughts. Yoga may prevent or decrease thoughts from becoming obsessions and thus help the individual master his or her compulsive behavior (30). Positive results have been found for yoga practice as a complementary treatment for medication or psychotherapy for General Anxiety Disorder and Obsessive-Compulsive Disorder, including for imprisoned individuals (24). Moreover, significant improvements in concentration (31), sustained attention (10), and memory (32) have been measured after yoga practice.

This study also shown that yoga, but not non-yoga free-choice exercise, can significantly reduce symptoms of somatization. Yoga, in addition to its positive effects on mental conditions such as anxiety and depression, has also been found to have a positive effect on somatization symptoms (33), which is in accordance with our findings. Clinical symptoms of somatization include dizziness, headache, chest pain, nausea, difficulty breathing, feelings of body weakness, body numbness, and a heavy feeling in the body. Through the different variations of body postures the participants of yoga classes increase their body awareness, thus improving their sense of control over their own body (31), their perception of bodily dysfunction, and their ability to prevent psychosomatic symptoms (33).

The above discussed results of our study could be explained by underlining neurobiological changes. For example several studies have investigated the effects of yoga on the sympathetic and parasympathetic functions of the autonomic nervous system as well as on the regulation of the hypothalamus-pituitary-adrenal axis (29, 34). These studies show that regular yoga exercise can reduce the levels of cortisol and catecholamines (adrenaline and noradrenaline) and increase the levels of serotonin, melatonin and gamma-amino butyric acid, which are all important factors in the regulation of mental health. Other physiological effects of yoga practice, in particular improved cortisol levels, can also be associated with improved self-esteem and mental and emotional states. These yoga-induced chemical changes in the body entail improved experiences of well-being and reduced levels of psychological distress (34).

### Strengths and limitations

The present study was a randomized controlled trial. This method is used to minimize the bias effects of extraneous or irrelevant variables on the measurements, and provides the strongest evidence of the effects of a treatment.

One limitation of the study is that the information about inmates' psychiatric health profile was operationalized and not assessed during a clinical examination. In addition, the use of self-reported measures is a limitation. Self-reported measures rely greatly on the respondent's capability to remember and admit the “truth”; answers may be distorted by social desirability and recall biases (35). However, self-reported research instruments present the significant advantages that they can be used without any clinical experience, are cost efficient, and can cover considerably larger numbers of individuals than when clinical measurements or semi-structured interviews are used for data collection in a given time frame. Furthermore, it has been found that in some cases it can be easier for respondents to report problems and complaints through an anonymous self-report, than during face-to-face clinical interview surveys or measures (35).

## Conclusion and suggestions for further research

This study confirms that physical activity has a positive effect on the psychological distress levels of prison inmates and provides new evidence about the specific positive effect of yoga on symptoms such as paranoid thoughts, memory problems, difficult decision making, trouble concentrating, obsessive thoughts, perception of bodily dysfunction, and somatization. The provision of opportunities for prison inmates to carry out physical activity in general and yoga exercise in particular can be an important tool for improving their reintegration into society and for helping them lead a drug free and law-abiding lifestyle upon release, which hypothesis should be tested in larger scale studies including follow-up recording of eventual long-term effects of the treatment.

## Author contributions

AS was responsible for data analysis and interpretation, continuous progress monitoring, manuscript drafting and submission. PM and ST contributed to data analysis, interpretation, and manuscript drafting. NK designed and led the project and supervised the drafting and development of the manuscript.

### Conflict of interest statement

NK was employed by the Swedish Prison and Probation Service during the study. The remaining authors declare that the research was conducted in the absence of any commercial or financial relationships that could be construed as a potential conflict of interest.

## References

[1] Homel R Thompson C Causes and prevention of violence in prisons. In: O'Toole S Eyland S editors. Corrections Criminology. Sydney, NSW: Hawkins Press (2005). p. 101–8.

[2] Slade K Samele C Valmaggia L Forrester A. Pathways through the criminal justice system for prisoners with acute and serious mental illness. J Forensic Leg Med. (2016) 44:162–8. 10.1016/j.jflm.2016.10.007 27810587

[3] Hofvander B Anckarsäter H Wallinius M Billstedt E. Mental health among young adults in prison: the importance of childhood-onset conduct disorder. Br J Psychiatr Open (2017) 3:78–84. 10.1192/bjpo.bp.116.003889 28357134 PMC5362727

[4] Markowitz FE Mental illness, crime, and violence: risk, context, and social control. Aggress Violent Behav. (2011) 16:36–44. 10.1016/j.avb.2010.10.003

[5] Fazel S Danesh J. Serious mental disorder in 23000 prisoners: a systematic review of 62 surveys. Lancet (2002) 359:545–50. 10.1016/S0140-6736(02)07740-1 11867106

[6] Sansone RA Sansone LA. Borderline personality and criminality. Psychiatry (2009) 6:16–20. 20011575 PMC2790397

[7] Moffitt TE Caspi A Harrington H Milne BJ. Males on the life-course-persistent and adolescence-limited antisocial pathways: Follow-up at age 26 years. Dev Psychopathol. (2002) 14:179–207. 10.1017/S0954579402001104 11893092

[8] Mohr-Jensen C Steinhausen HC. A meta-analysis and systematic review of the risks associated with childhood attention-deficit hyperactivity disorder on long-term outcome of arrests, convictions, and incarcerations. Clin Psychol Rev. (2016) 48:32–42. 10.1016/j.cpr.2016.05.002 27390061

[9] Kriminalvården Rehabilitation - Swedish Prison and Probation Service | Kriminalvården (2018) Available online at: https://www.kriminalvarden.se/swedish-prison-and-probation-service/rehabilitation/ (Accessed April 21, 2018).

[10] Kerekes N Fielding C Apelqvist S. Yoga in correctional settings: a randomized controlled study. Front Psychiatry (2017) 8:204. 10.3389/fpsyt.2017.00204 29085307 PMC5650609

[11] Sharma M Haider T Yoga as an alternative and complementary therapy for patients suffering from anxiety. J Evid Based Complement Altern Med. (2013) 18:15–22. 10.1177/2156587212460046 PMC5871178 26787730

[12] Kahya HH Raspin CG. Yoga therapy for the mind eight-week course: participants' experiences. Explor J Sci Heal. (2017) 13:116–23. 10.1016/j.explore.2016.12.006 28131764

[13] Streeter CC Gerbarg PL Saper RB Ciraulo DA Brown RP. Effects of yoga on the autonomic nervous system, gamma-aminobutyric-acid, and allostasis in epilepsy, depression, and post-traumatic stress disorder. Med Hypotheses (2012) 78:571–9. 10.1016/j.mehy.2012.01.021 22365651

[14] Rani K Tiwari S Singh U Agrawal G Ghildiyal A Srivastava N. Impact of yoga nidra on psychological general wellbeing in patients with menstrual irregularities: a randomized controlled trial. Int J Yoga (2011) 4:20–5. 10.4103/0973-6131.78176 21654971 PMC3099097

[15] Michalsen A Jeitler M Brunnhuber S Lüdtke R Büssing A Musial F. Iyengar yoga for distressed women: a 3-armed randomized controlled trial. Evid Based Complement Altern Med. (2012) 2012:408727. 10.1155/2012/408727 23049608 PMC3463199

[16] Raghuram N Deshpande S Nagendra H. A randomized control trial of the effect of yoga on verbal aggressiveness in normal healthy volunteers. Int J Yoga (2008) 1:76–82. 10.4103/0973-6131.41034 21829289 PMC3144615

[17] Bilderbeck AC Farias M Brazil IA Jakobowitz S Wikholm C. Participation in a 10-week course of yoga improves behavioural control and decreases psychological distress in a prison population. J Psychiatr Res. (2013) 47:1438–45. 10.1016/j.jpsychires.2013.06.014 23866738

[18] Klatte R Pabst S Beelmann A Rosendahl JS. The efficacy of body-oriented yoga in mental disorders. Dtsch Arztebl Int. (2016) 113:195–202. 10.3238/arztebl.2016.0195 27118717 PMC5400032

[19] Jeter PE Slutsky J Singh N Khalsa SBS. Yoga as a therapeutic intervention: a bibliometric analysis of published research studies from 1967 to 2013. J Altern Complement Med. (2015) 21:586–92. 10.1089/acm.2015.0057 26196166 PMC4605382

[20] de Manincor M Bensoussan A Smith CA Barr K Schweickle M Donoghoe LL. Individualized yoga for reducing depression and anxiety, and improving well-being: a randomized controlled trial. Depress Anxiety (2016) 33:816–28. 10.1002/da.22502 27030303

[21] Derogatis LR. The brief symptom inventory: an introductory report. Psychol Med. (1983) 13:595–605. 10.1017/S0033291700048017 6622612

[22] Cohen J Statistical Power Analysis for the Behavioral Sciences. 2nd ed. Hillsdale, NJ: Erlbaum (1988).

[23] Ströhle A. Physical activity, exercise, depression and anxiety disorders. J Neural Transm. (2009) 116:777–84. 10.1007/s00702-008-0092-x 18726137

[24] Battaglia C Di Cagno A Fiorilli G Giombini A Borrione P Baralla F. Participation in a 9-month selected physical exercise programme enhances psychological well-being in a prison population. Crim Behav Ment Heal. (2015) 25:343–54. 10.1002/cbm.1922 25106026

[25] Tolbaños Roche L Miró Barrachina MT Ibáñez Fernández I Effect of “Exercise Without Movement” yoga method on mindfulness, anxiety and depression. Complement Ther Clin Pract. (2016) 25:136–41. 10.1016/j.ctcp.2016.09.008 27863603

[26] Balasubramaniam M Telles S Doraiswamy PM. Yoga on our minds: a systematic review of yoga for neuropsychiatric disorders. Front Psychiatry (2013) 3:117. 10.3389/fpsyt.2012.00117 23355825 PMC3555015

[27] Vancampfort D De Hert M Vansteenkiste M De Herdt A Scheewe TW Soundy A. The importance of self-determined motivation towards physical activity in patients with schizophrenia. Psychiatry Res. (2013) 210:812–8. 10.1016/j.psychres.2013.10.004 24182688

[28] Visceglia E Lewis S. Yoga therapy as an adjunctive treatment for schizophrenia: a randomized, controlled pilot study. J Altern Complement Med. (2011) 17:601–7. 10.1089/acm.2010.0075 21711202

[29] Kirkwood G Rampes H Tuffrey V Richardson J Pilkington K Ramaratnam S. Yoga for anxiety: a systematic review of the research evidence. Br J Sports Med. (2005) 39:884–91; discussion: 891. 10.1136/bjsm.2005.018069 16306493 PMC1725091

[30] Bhat S Varambally S Karmani S Govindaraj R Gangadhar BN. Designing and validation of a yoga-based intervention for obsessive compulsive disorder. Int Rev Psychiatry (2016) 28:327–33. 10.3109/09540261.2016.1170001 27117898

[31] Javnbakht M Hejazi Kenari R Ghasemi M. Effects of yoga on depression and anxiety of women. Complement Ther Clin Pract. (2009) 15:102–4. 10.1016/j.ctcp.2009.01.003 19341989

[32] Rocha KKF Ribeiro AM Rocha KCF Sousa MBC Albuquerque FS Ribeiro S Improvement in physiological and psychological parameters after 6months of yoga practice. Conscious Cogn. (2012) 21:843–50. 10.1016/j.concog.2012.01.014 22342535

[33] Yoshihara K Hiramoto T Oka T Kubo C Sudo N. Effect of 12 weeks of yoga training on the somatization, psychological symptoms, and stress-related biomarkers of healthy women. Biopsychosoc Med. (2014) 8:1. 10.1186/1751-0759-8-1 24383884 PMC3892034

[34] Woolery A Myers H Sternlieb B Zeltzer L. A yoga intervention for young adults with elevated symptoms of depression. Altern Ther Health Med. (2004) 10:60–3. 15055096

[35] Nijman H Bjørkly S Palmstierna T Almvik R Assessing aggression of psychiatric patients: methods of measurement and its prevalence. In: Richter D Whittington R editors. Violence in Mental Health Settings - Causes, Consequences, Management. New York, NY: Springer (2006). p. 11–23.

